# Effect of Temperature and Cell Viability on Uranium Biomineralization by the Uranium Mine Isolate *Penicillium simplicissimum*

**DOI:** 10.3389/fmicb.2021.802926

**Published:** 2021-12-22

**Authors:** Sebastian Schaefer, Robin Steudtner, René Hübner, Evelyn Krawczyk-Bärsch, Mohamed L. Merroun

**Affiliations:** ^1^Institute of Resource Ecology, Helmholtz-Zentrum Dresden-Rossendorf, Dresden, Germany; ^2^Institute of Ion Beam Physics and Materials Research, Helmholtz-Zentrum Dresden-Rossendorf, Dresden, Germany; ^3^Department of Microbiology, University of Granada, Granada, Spain

**Keywords:** biomineralization, bioremediation, fungal biomass, uranium, waste water, *Penicillium simplicissimum*

## Abstract

The remediation of heavy-metal-contaminated sites represents a serious environmental problem worldwide. Currently, cost- and time-intensive chemical treatments are usually performed. Bioremediation by heavy-metal-tolerant microorganisms is considered a more eco-friendly and comparatively cheap alternative. The fungus *Penicillium simplicissimum* KS1, isolated from the flooding water of a former uranium (U) mine in Germany, shows promising U bioremediation potential mainly through biomineralization. The adaption of *P. simplicissimum* KS1 to heavy-metal-contaminated sites is indicated by an increased U removal capacity of up to 550 mg U per g dry biomass, compared to the non-heavy-metal-exposed *P. simplicissimum* reference strain DSM 62867 (200 mg U per g dry biomass). In addition, the effect of temperature and cell viability of *P. simplicissimum* KS1 on U biomineralization was investigated. While viable cells at 30°C removed U mainly extracellularly *via* metabolism-dependent biomineralization, a decrease in temperature to 4°C or use of dead-autoclaved cells at 30°C revealed increased occurrence of passive biosorption and bioaccumulation, as confirmed by scanning transmission electron microscopy. The precipitated U species were assigned to uranyl phosphates with a structure similar to that of autunite, *via* cryo-time-resolved laser fluorescence spectroscopy. The major involvement of phosphates in U precipitation by *P. simplicissimum* KS1 was additionally supported by the observation of increased phosphatase activity for viable cells at 30°C. Furthermore, viable cells actively secreted small molecules, most likely phosphorylated amino acids, which interacted with U in the supernatant and were not detected in experiments with dead-autoclaved cells. Our study provides new insights into the influence of temperature and cell viability on U phosphate biomineralization by fungi, and furthermore highlight the potential use of *P. simplicissimum* KS1 particularly for U bioremediation purposes.

**Graphical Abstract:**
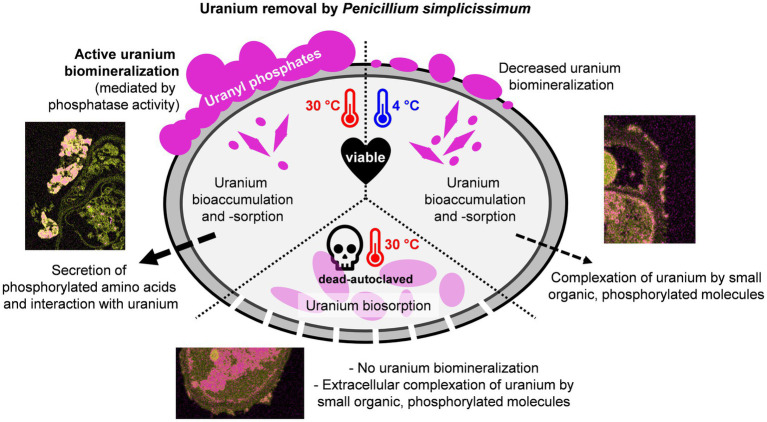

## Introduction

As a result of former uranium (U) mining and milling activities, large amounts of wastewater containing high concentrations of U and other heavy metals have been generated, with the potential risk of contaminating the surrounding environment. Once disposed into the environment, U could eventually reach the top of the food chain and be ingested by humans, causing health risks like severe kidney and liver damage (Keith et al., 2013). Therefore, it is necessary not only to clean up contaminated sites, but also to treat U-contaminated wastewater in order to prevent heavy metal release to the environment. The former U mine in Königstein (Germany) represents such a contaminated site. Between 1984 and 1990, the radionuclide U was extracted from the rock material, mainly composed of sandstone, by *in-situ* leaching – i.e., injection of sulfuric acid into the underground rock. The resulting U-bearing, acidic liquid was collected and further processed to finally recover the heavy metal (Zeißler et al., 2006). Since the closure of U mining activities in Germany, the sub-surface of the mine has been remediated by controlled flooding to prevent the contamination of aquifers. At present, the flooding water is still characterized by relatively high concentrations of U (~8–9 mg/L) and a low pH of 2.9 owing to the acidic leaching process (Kassahun et al., 2015). Furthermore, the concentration of heavy metals like cadmium, nickel, and zinc are elevated (Zirnstein, 2015). The water consequently has to be pumped to the surface and is currently treated by a conventional, chemical wastewater treatment plant.

Such chemical treatments are time- and cost-intensive, however (Azubuike et al., 2016; Verma and Kuila, 2019). Depending on the on-site situation, chemistry-based techniques often generate hazardous waste and become less efficient at decreasing pollutant concentrations (Azubuike et al., 2016; Verma and Kuila, 2019). For several years, science has been concerned with alternative bioremediation approaches. Bioremediation aims to use suitable microorganisms to prospectively support or outperform chemical treatment. Microorganisms used in bioremediation should fulfill several criteria including: (i) high tolerance to heavy metals and radionuclides; (ii) metabolic versatility; and (iii) ability to reduce solubility and mobility of the inorganic contaminants. Microbial interaction mechanisms with heavy metals are mainly clustered into passive and active processes, based on their dependence on active cell metabolism. In the passive biosorption process, the cationic heavy metal, for example U(VI), binds to components of the fungal cell wall, e.g., phosphorylated polysaccharides and intracellularly to negatively charged functional groups like phosphate or carbonate groups (Tsezos and Volesky, 1982; González-Muñoz et al., 1997; Lloyd and Macaskie, 2002; Limcharoensuk et al., 2015; Kulkarni et al., 2016; Bano et al., 2018; Lopez-Fernandez et al., 2018; Segretin et al., 2018). Active microbial interaction mechanisms are further subdivided into anaerobic enzymatic reduction, biomineralization, and bioaccumulation. Bioaccumulation describes the active, controlled uptake of heavy metals (e.g., *via* siderophores) and their subsequent intracellular precipitation, which is still under investigation (Limcharoensuk et al., 2015; Gerber et al., 2018; Segretin et al., 2018). Microorganisms can also secrete negatively charged metabolites, such as hydrogen phosphates, hydrogen carbonates, oxalates, or hydroxides. This process is called biomineralization and leads to extracellular precipitation and detoxification (Merroun et al., 2011; Kaewdoung et al., 2016; Chandwadkar et al., 2018).

Besides bacteria, various fungal species have been detected at uranium mining sites and are known for their elevated heavy-metal adaption and tolerance (De Silóniz et al., 2002; Anahid et al., 2011; Zirnstein et al., 2012; Gerber et al., 2018; Glukhova et al., 2018; Stępniewska et al., 2020; Coelho et al., 2020a). Therefore, they are considered as putative candidates for bioremediation approaches to remove heavy metals from contaminated soil or wastewater (Song et al., 2019; Coelho et al., 2020b). In terms of fungal biomineralization and biosorption of U, phosphates and extracellular phosphatase activity have been reported to be the key players (Liu et al., 2010; Günther et al., 2014; Liang et al., 2015, 2016; Vázquez-Campos et al., 2015; Zheng et al., 2017; Wollenberg et al., 2021). While different physico-chemical parameters like pH or background medium composition have been assessed for their influence on the biomineralization of U, the impact of metabolic activity is not yet fully investigated. In the present study, the fungal strain *Penicillium simplicissimum*, isolated from the flooding water of the former U mine in Königstein (Germany), was investigated toward its potential use for bioremediation purposes, particularly for U-contaminated sites. We focused on changes in the uranium bioremoval by *P. simplicissimum* KS1 depending on temperature and cell viability to unravel metabolic reliance. Additionally, the ability of *P. simplicissimum* KS1 to effectively remove U was compared to the *P. simplicissimum* reference strain DSM 62867 to investigate an adaption to U-contaminated environments.

## Materials and Methods

### Microorganisms and Culture Conditions

The fungus *P. simplicissimum* KS1 was isolated from the flooding water of the former U mine in Königstein (Germany) by culture-dependent methods using Sabouraud-Dextrose (SD, bacto-peptone 5.0 g/L, casein peptone 5.0 g/L, glucose 40.0 g/L, and Carl Roth) medium (Gerber et al., 2015), adapted from Odds (1991). As a comparative fungal species, *P. simplicissimum* DSM 62867 was purchased from DSMZ (Leibniz Institute DSMZ-German Collection of Microorganisms and Cell Cultures). Both strains were grown in SD medium at 30°C and 130 rpm (Thermoshake EA2, C. Gerhardt) for 72 h and stored at 4°C on SD agar plates after growth at 30°C for 72 h.

### DNA Isolation and Sanger Sequencing of the Fungal Isolate KS1

The DNA of KS1 was isolated by following the protocol for alkaline DNA extraction (Birnboim and Doly, 1979). A purification and concentration step were performed according to the instructions of the DNA Clean & Concentrator™-5 Kit (Zymo Research). A fungal-characteristic DNA fragment of the internal transcribed spacer (ITS) region of the 18S rRNA gene was amplified by PCR using the primers ITS5 and ITS4 (both Thermo Fisher Scientific), according to Martin and Rygiewicz (2005). The obtained PCR products were purified (DNA Clean & Concentrator™-5 Kit) and sequenced by Sanger sequencing performed by GATC Biotech. The obtained sequences were aligned and compared to those in the nucleotide-nucleotide Basic Local Alignment Search Tool (blastn) database of the National Center for Biotechnology Information (NCBI).^1^ The Sanger sequencing results are available on NCBI GenBank® under accession number SAMN22830865.

### Fungal U Removal Capacity Studies

To investigate the removal capacity of U, the fungal cells were grown in SD medium for 72 h. Afterwards the cells were separated from the medium and washed twice by sterile filtration and resuspension in sterile-filtered tap water (pH = 5.0). Five milliliters culture were subsequently diluted in 45 ml sterile-filtered tap water (pH = 5.0) to reach a final dry biomass (DBM) of 0.10 ± 0.02 g/L. A uranyl stock solution [UO_2_(NO_3_)_2_] was added to a final concentration of 0.1 mM. The samples were incubated for 52 h with agitation at 130 rpm at 4 and 30°C, using pre-tempered chemicals. Sterile-filtered samples, each with a volume of 500 μl, were regularly taken. To each sample, 5 μl of concentrated nitric acid was added immediately. The samples were stored at 4°C and used for determination of the U concentration by means of inductively coupled plasma mass spectrometry (ICP-MS) using a NexION 350X (PerkinElmer). To determine the effect of cell viability on U removal, grown fungal cells in SD medium were autoclaved for 30 min at 121°C. The autoclaved cell culture was centrifuged and washed twice with sterile-filtered tap water (pH = 5.0), then further treated as described above. The DBM was determined after performing the respective experiment. Thereby, cells were separated from the medium by sterile filtration on a pre-dried, weighed filter. The biomass on the filter was subsequently dried overnight at 80°C before final weighing.

### Determination of Orthophosphate Concentration and Acid-Phosphatase Activity

In order to determine the orthophosphate concentration and the acid-phosphatase activity involved in fungal U removal, washed fungal cells (DBM 0.10 ± 0.02 g/L) were either suspended in 100 ml SD medium or in 100 ml sterile-filtered tap water (pH = 5.0). The cells in SD medium were incubated for 52 h at 30°C and 130 rpm. The cells in sterile-filtered tap water (pH = 5.0) were further prepared and incubated as described in section “Fungal U Removal Capacity Studies.” Samples of each experiment were sterile-filtered after 52 h, and 1 ml of each sample was analyzed for its orthophosphate concentration. To this end, an ion chromatograph system Dionex™ Integrion™ HPIC™ (Thermo Fisher Scientific) was utilized with the following equipment: analytical column (Dionex IonPac, AS23 – 4 μm, RFIC, 2x 250mm), guard column (Dionex IonPac, AG23 – 4 μm, RFIC, 2x 50 mm), and eluent 4.5 mM Na_2_CO_3_/0.8 mM NaHCO_3_. Additionally, 1 ml of the samples was analyzed for its acid-phosphatase activity following the instructions of the Acid Phosphatase Activity Fluorometric Assay Kit (Sigma-Aldrich): 200 μl of each sample and control solution were pipetted in a 96-well plate and analyzed for fluorescence using the microplate luminescence reader Mithras 2 (Berthold Technologies), equipped with 355 × 40 excitation and 460 × 25 emission filter, for 30 s with a counting time of 0.1 s and a lamp energy of 40%. All experiments were performed in triplicate.

### Scanning Electron Microscopy

For SEM measurements, fungal cells of the two *P. simplicissimum* strains KS1 and DSM 62867 were treated with U for 52 h, as described in section “Fungal U Removal Capacity Studies.” The cells were recovered by centrifugation (10 min, 13,793 *g*, 4°C). The supernatant was removed, and the pellet was further processed for SEM at the *Centro de Instrumentación Científica* (University of Granada, Spain), according to Anderson (1951). The specimens were imaged using a S-4800 microscope (Hitachi) operated at an accelerating voltage of 10 kV. For qualitative chemical analysis, energy-dispersive X-ray spectroscopy (EDXS) was carried out at 30 keV using a conventional Si(Li) detector with a S-UTW window. Additional studies were performed using a GEMINI FESEM microscope (Carl Zeiss) operated at an accelerating voltage of 20 kV.

### High-Angle Annular Dark-Field Scanning Transmission Electron Microscopy (HAADF-STEM)

For HAADF-STEM measurements, U interaction experiments with *P. simplicissimum* KS1 or DSM 62867 were performed at 30°C and, in the case of *P. simplicissimum* KS1, additionally at 4 and 30°C with autoclaved, non-viable cells (as described in section “Fungal U Removal Capacity Studies”). After 52 h, the cells were immediately centrifuged (10 min, 13,793 *g*, 4°C). The supernatant was removed, the pellet was washed three times with sterile-filtered tap water (pH = 5.0) and subsequently fixed with glutardialdehyde at 1% (v/v) from 50% stock solution (v/v) and stored at 4°C. The *P. simplicissimum* DSM 62867 sample was further processed for STEM analysis at the *Centro de Instrumentación Científica* (University of Granada, Spain), according to Renau Piqueras and Megias Megias (1998). *Penicillium simplicissimum* KS1 samples were further prepared at the Advanced Imaging/Electron Microscopy facility of the Center for Molecular and Cellular Bioengineering (Technische Universität Dresden, Germany). HAADF-STEM imaging and spectrum imaging analysis based on EDXS were performed at 200 kV with a Talos F200X microscope equipped with an X-FEG electron source and a Super-X EDX detector system (FEI). Prior to STEM analysis, the specimen – mounted on a high-visibility low-background holder – was placed for 2 s inside Model 1,020 Plasma Cleaner (E. A. Fischione Instruments Inc.).

### Cryo-TRLFS Measurements

For the determination of potential U(VI) species formed by the *P. simplicissimum* strain KS1, time-resolved laser-induced fluorescence spectroscopy (TRLFS) was used. The detection limit for aqueous U is currently 0.2 μg/L (Bernhard and Geipel, 2007). The cryo-TRLFS samples were prepared as described in section “Fungal U Removal Capacity Studies” using a fungal DBM of around 0.25 g/L. Thereby, *P. simplicissimum* KS1 was studied in the presence of 0.1 mM U(VI) at 4 and 30°C. Additionally, autoclaved cells were investigated at 30°C at an initial U(VI) concentration of 0.1 mM. As control samples, *P. simplicissimum* KS1 was prepared without U(VI), and 0.1 mM U(VI) solutions without fungal biomass were measured after an incubation at 30°C. All samples were incubated for 48 h. After the interaction experiments, the cell pellets were separated from the supernatant by centrifugation at 5445 *g* for 20 min. The pellets were washed twice with sterilized tap water (pH 5.0), and both supernatant and fungal biomass were separately shock-frozen in plastic cuvettes by liquid nitrogen and stored at −80°C. The U(VI) luminescence at 153 K was measured after excitation with laser pulses at 266 nm (Minilite high-energy solid-state laser; Continuum) and an average pulse energy of 300 mJ. The emission of the samples was recorded using an iHR550 spectrograph (HORIBA Jobin Yvon) and an ICCD camera (HORIBA Jobin Yvon) in the 425.0–625.0 nm wavelength range by averaging 100 laser pulses and using a gate time of 10 ms. TRLFS spectra were analyzed and deconvoluted by means of parallel factor analysis (PARAFAC) using the N-way toolbox with Matlab R2015a (Andersson and Bro, 2000). PARAFAC is known to be a valuable tool for luminescence data deconvolution, since PARAFAC data processing delivers information about speciation, individual emission spectra, and luminescence decays in both chemical and biological systems (Bader et al., 2016, 2019; Drobot et al., 2016).

## Results and Discussion

### Isolation and Physiological Characterization of the Fungal Isolate *Penicillium simplicissimum* KS1

Using culture-dependent methods, *P. simplicissimum* KS1 was previously isolated on SD medium from the flooding water of the former U mine in Königstein (Germany; Gerber et al., 2015). Compared to the other isolated eukaryotic and prokaryotic strains, *P. simplicissimum* KS1 displayed a high U removal capacity (Gerber et al., 2015) and was therefore chosen for further studies. By sequencing the ITS 18S rRNA gene and comparison with blastn (NCBI), *P. simplicissimum* KS1 (accession: SAMN22830865) displayed a maximum phylogenetic identity with *P. simplicissimum* (accession: MH856014.1; 100% query cover; 98.49% identity) and the taxonomical synonymous *Penicillium pulvilorum* (accession: KF624805.1; 100% query cover; 98.35% identity). The microbial diversity in the flooding water is known to be dominated by iron- and sulfur-oxidizing bacteria, as well as iron-reducing bacteria (Zirnstein, 2015; Gerber, 2019). However, archaea and eukaryotes, including fungal species, were detected as well (Zirnstein et al., 2012; Zirnstein, 2015; Gerber, 2019). Previously, our group isolated another heavy metal-tolerant fungal species from the flooding water of Königstein that belongs to the division of Basidiomycota (Gerber et al., 2018) – in contrast to *P. simplicissimum* KS1, which is an ascomycetous fungus.

To further characterize the fungal isolate, suitable carbon sources for the enrichment of the fungus were investigated (Supplementary Table 1). *Penicillium simplicissimum* KS1 showed good growth in the presence of glucose and fructose, and medium growth with galactose, mannose, saccharose, and xylose, whereas no growth was observed in ethanol, lactate, oxalic acid, and sodium acetate. The total organic content of the flooding water at the mining site in Königstein is below 1 mg/L (Zirnstein, 2015). It would therefore have to be enriched with carbon sources and the biomass itself for *in-situ* bioremediation. Alternatively, the presence and growth of microorganisms in the flooding water may be obtained through the biodegradation of underground wood constructions, which leads to a decomposition into mono- and polysaccharides (e.g., arabinose, glucose, xylose, and galactose; Baraniak et al., 2002).

In addition, the tolerance of *P. simplicissimum* KS1 toward selected heavy metals in solution was studied to evaluate its suitability for bioremediation applications (Supplementary Table 2). The highest tolerance was observed toward chromium (>22 mM) and zinc (>15 mM), whereas nickel and U inhibited the growth of *P. simplicissimum* KS1 at concentrations of 0.2 and 0.7 mM, respectively. The highest toxicity of nickel but lower for zinc is in good agreement with the observations of Anahid et al. (2011). The reported distinctly higher tolerance concentrations by Anahid et al. (2011) might be caused by the utilization of a *P. simplicissimum* strain that could be more tolerant to heavy metals due to (i) a potentially artificially increased heavy-metal adaption of the fungal strain *via* sub-culturing prior to the metal tolerance test or (ii) a naturally stronger adaption to heavy metal – e.g., due to a heavy-metal-exposed place of origin. Also, the heavy-metal tolerance was investigated on solid media in contrast to liquid media which was used in the present work. The fungal yeast *Rhodosporidium toruloides* was also isolated from the flooding water of the former U mine in Königstein and likewise showed elevated heavy-metal tolerance (Gerber et al., 2018). While this fungal strain was more tolerant toward U (up to 6 mM), its tolerance toward chromium, copper, cadmium, and zinc is low compared to *P. simplicissimum* KS1 (Gerber et al., 2018). This finding highlights the suitability of *P. simplicissimum* KS1 for bioremediation purposes involving various heavy metals.

### U Bio-Association Studies: Effect of Temperature and Cell Viability

**Figure 1:**
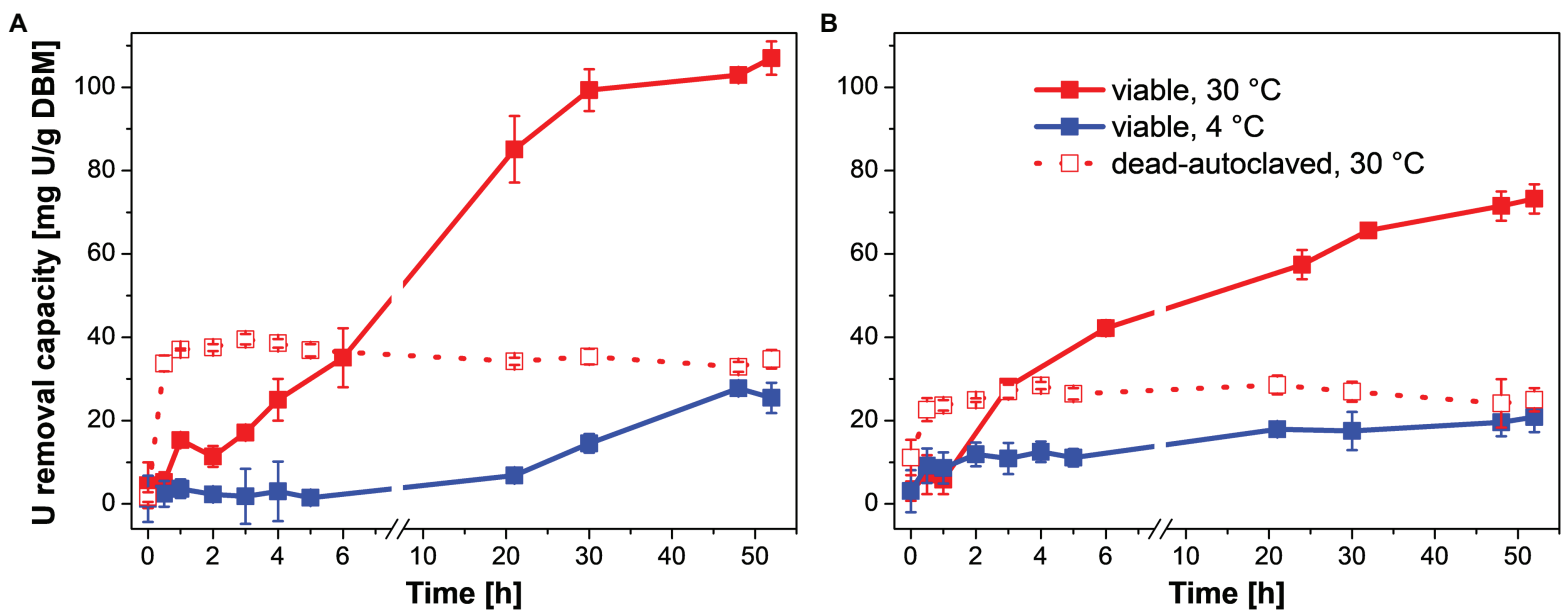
U removal capacity of *Penicillium simplicissimum* KS1 **(A)** and DSM 62867 **(B)** at ~0.1 g DBM/L over 52 h at 4°C (blue) and 30°C (red) and 0.1 mM initial U concentration. At 30°C, viable (straight line) and dead-autoclaved cells (dotted line) were studied. SDs are depicted as error bars.

The influence of temperature and cell viability on the U removal capacity of *P. simplicissimum* KS1 and the *P. simplicissimum* reference strain DSM 62867 was investigated (Figure 1). *Penicillium simplicissimum* DSM 62867 was selected as a most likely reference strain that is not heavy-metal-adapted; since contrary to *P. simplicissimum* KS1, the strain was isolated from pristine soil samples in Germany. Kinetic U removal studies at different temperatures (4 and 30°C) and cell viability over 52 h suggested a three-phase U removal for both fungal strains (Figure 1). The U concentration was set to 0.1 mM, representing the U concentration that could emerge in the mining site resulting from a prospectively envisaged rise of flooding levels. Currently, the U concentration in Königstein (Germany) ranges between ~0.03 and 0.04 mM. Two temperatures were chosen: the optimal growth temperature for fungal species (30°C), plus a lower temperature (4°C), so as to study a possible metabolic influence on U interaction.

First, U may have been removed passively by biosorption of *P. simplicissimum* as indicated by a linear increase in U removal during the first 5 h at 30°C (Figure 1, straight red line). This linear increase was followed by a less-steep increase in U removal at 30°C up to 24–30 h of incubation. This second phase was significantly reduced with a temperature decrease to 4°C (Figure 1, blue line) demonstrating active metabolic processes additionally involved in passive biosorption during the U removal at 30°C (viable *P. simplicissimum* KS1 30°C: 107 mg U/g DBM, 4°C: 27 mg U/g DBM; viable *P. simplicissimum* DSM 62867 30°C: 72 mg U/g DBM, 4°C: 20 mg U/g DBM). Furthermore, a decline in apparent cell viability after 24 h at 30°C was observed for *P. simplicissimum* KS1 (Supplementary Figure 1), which overlaps with the decrease in slope of U removal capacity, as well as a plateau after around 30 h. This suggests a third process entailing passive U biosorption of dead fungal biomass (Pang et al., 2011). Similar U removal processes were reported for other U-tolerant fungal species, driven by active bioaccumulation and passive biosorption (Gerber et al., 2018; Wollenberg et al., 2021).

To support our hypothesis that active metabolic processes are involved in U removal by *P. simplicissimum* KS1 and DSM 62867, the U removal capacity of dead-autoclaved fungal biomass at 30°C was also studied (Figure 1, dotted red lines). Thus, viable cells revealed higher U accumulation values compared to those of dead-autoclaved cells after 2 days (*P. simplicissimum* KS1 viable: 107 mg U/g DBM, dead: 34 mg U/g DBM; *P. simplicissimum* DSM 62867 viable: 72 mg U/g DBM, dead: 24 mg U/g DBM). U removal by dead cells is driven by immediate passive biosorption, as reported for various fungal species (Pang et al., 2011; Gerber et al., 2018; Wollenberg et al., 2021); and it can even surpass the U removal of viable cells *via* sorption to released or exposed compounds upon cell death, like lipopolysaccharides or phosphates, as seen for *Coniochaeta fodinicola* (Vázquez-Campos et al., 2015). Our observation implies a more prominent involvement of active metabolic processes in the U removal of *P. simplicissimum* KS1 – for instance biomineralization and intracellular accumulation – than passive biosorption. For both strains, dead-autoclaved fungal biomass removed more U from the solution when compared to viable cells at 4°C, which may be explained by additional available binding sites, both intra- and extracellularly, due to damaged cell walls.

Moreover, *P. simplicissimum* KS1 removed more U from the solution than *P. simplicissimum* DSM 62867 under similar environmental conditions (Figure 1) and *P. simplicissimum* KS1 showed a slower response to the U stress at 4°C. Both these observations support an adaption of the fungal isolate to heavy-metal stress, as compared to the reference strain, which was not exposed to heavy metals before isolation.

Remarkably, *P. simplicissimum* KS1 was able to remove up to 80% of the initially introduced U from solution after 48 h, depending on the fungal biomass concentration (Supplementary Figure 2). With an increase in fungal biomass from 0.05 to 0.58 g/L, an exponential decrease in U removal capacity (normalized by the actual biomass) was observed for both *P. simplicissimum* KS1 and DSM 62867. Overall, *P. simplicissimum* DSM 62867 removed less U than *P. simplicissimum* KS1, especially for a biomass of around 0.1 g/L and lower, as can be seen in Figure 1 for a fixed DBM around 0.1 g/L. The maximum U removal capacity of *P. simplicissimum* KS1 of ~550 mg U/g DBM outperformed not only the reference strain *P. simplicissimum* DSM 62867 (~200 mg U/g DBM), but also other fungal species including *Saccharomyces cerevisiae*, *Rhizopus* sp., and *R. toruloides* (Supplementary Table 3), which again proves its great potential for bioremediation purposes. However, a direct comparison of those values is difficult; the experimental conditions vary between different studies and, especially, the physicochemical conditions of the respective experimental setup (pH, temperature, or biomass concentration) are known to tremendously affect the U removal capacity of microorganisms (Bustard et al., 1997; Gerber et al., 2018; Zheng et al., 2018). For this reason, only the maximum U removal capacities, observed respectively, are compared in Supplementary Table 3.

### HAADF-STEM Characterization of U Biomineralization by *P. simplicissimum* KS1 Cells

**Figure 2:**
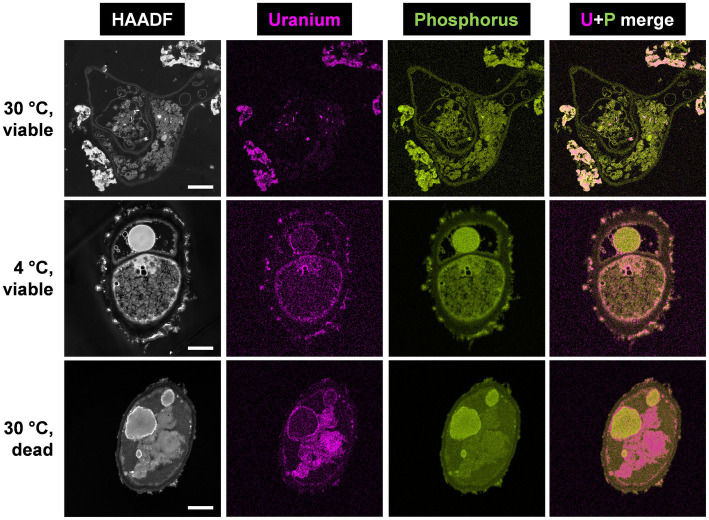
HAADF-STEM micrographs of viable *P. simplicissimum* KS1 at 30 and 4°C (top and center rows) and dead-autoclaved cells at 30°C (bottom row) together with EDXS-based element distributions for uranium (magenta) and phosphorus (green). The fungal isolate was incubated in 0.1 mM U (background electrolyte: sterile-filtered tap water pH 5.0) for 48 h. The scale bars indicate 1 μm.

HAADF-STEM imaging combined with EDXS-based element distribution analysis was performed to investigate the effect of temperature and cell viability on the cellular localization of U complexes and the underlying interaction mechanisms of U with the fungus *P. simplicissimum* KS1 (Figure 2). Metabolically active fungal cells were incubated with 0.1 mM U for 48 h at 4 and 30°C, in addition to dead-autoclaved cells, which were only incubated at 30°C for the same time and at the same U concentration.

Spectrum imaging analysis of the samples showed significant differences in the amount of accumulated U and its cellular localization. For metabolically active and viable cells at 30°C (Figure 2, top row), large extracellular U precipitations were detected, in addition to low intracellular amounts of U accumulations. The extracellular U precipitations showed an amorphous nature, and thus differed structurally from the needle-like objects observed intracellularly. Additional SEM studies combined with EDXS analysis (Supplementary Figures 3, 4) confirmed that the removed U is localized extracellularly by *P. simplicissimum* KS1 (and DSM 62867). The structure of the U accumulations evoked a biomineralization-mediated precipitation (Liang et al., 2015).

With a decrease in temperature to 4°C (Figure 2, center row), *P. simplicissimum* KS1 appeared to accumulate U at the cell surface and intracellularly. Large extracellular accumulations, as detected at 30°C, were not observed at 4°C. These results indicate that the large extracellular U accumulations were driven by a metabolically active process, i.e., biomineralization. Biomineralization relies on the activity of enzymes, such as phosphatases, to degrade organic phosphates, giving rise to the generation of orthophosphate. Biomineralization is therefore barely observable at lower temperatures and metabolically inactive cells (Liang et al., 2015). Due to cell death after 24 h at 30°C and putative damage to the fungal cell wall, U perhaps entered the cells; this would have been followed by passive biosorption by negatively charged functional groups and might have been bound to the release of cellular compounds, plus cell wall, and membrane fragments (Vázquez-Campos et al., 2015).

Inactivation of *P. simplicissimum* KS1 by autoclavation, with incubation at 30°C (Figure 2, bottom row), led to U precipitations that were mainly visible intracellularly, along with minor U biosorption at the cell surface. Considering the control samples of untreated viable and untreated dead-autoclaved cells (Supplementary Figure 5), the dead-autoclaved cells showed partial detachments of the cell wall, possibly offering additional binding sites for U and facilitating the influx of the heavy metal and subsequent passive intracellular U biosorption. The use of dead fungal biomass for bioremediation of U-contaminated wastewater is an alternative approach (Vázquez-Campos et al., 2015; Coelho et al., 2020b). Under the experimental conditions chosen by Vázquez-Campos et al. (2015), dead fungal biomass removed more U compared to the viable cells. Yet, other fungal species – *P. simplicissimum* KS1 (Figure 1) in the present work, or *R. toruloides* (Gerber et al., 2018) – removed elevated amounts of U by viable cells. Hence, the fungal isolate *P. simplicissimum* KS1 could represent a source for both bioremediation approaches to remove U from wastewater – exploiting viable or dead cells.

EDXS-based element mapping in Figure 2 revealed U association with phosphorus, which indicates biomineralization and biosorption of U phosphates extra- and intracellularly. The contribution of phosphorus in U bioprecipitation has been previously reported (Liu et al., 2010; Günther et al., 2014; Liang et al., 2015, 2016; Vázquez-Campos et al., 2015; Zheng et al., 2017; Wollenberg et al., 2021). However, phosphorus was not solely detected superimposed upon the U signal (Supplementary Figure 6). Other elements, such as nitrogen, could be explained by putative biosorption of U by biopolymers (for example, chitin, cellulose and its derivatives) after damaging the fungal cell wall and passive biosorption of amino functionalities (Galun et al., 1984; Zhao et al., 2016).

For comparison, *P. simplicissimum* DSM 62867 and its interaction with U at 30°C were studied by means of HAADF-STEM and SEM as well (Supplementary Figures 4, 7). Similar to *P. simplicissimum* KS1, SEM revealed extracellular U biomineralization, although spectrum imaging analysis displayed some minor differences. Most notably, the amount of intracellular U accumulation increased significantly. The differences between *P. simplicissimum* KS1 and DSM 62867 indicated an adaptation of *P. simplicissimum* KS1 to U. Uranium is not as prominently present intracellularly in the fungal isolate, which may have adapted its metabolic response to heavy metal environmental stress, as observed previously in increased heavy metal resistance and U removal from solution.

### Determination of Extracellular Orthophosphate and Phosphatase Activity of *P. simplicissimum* KS1 in the Presence of U

**Figure 3:**
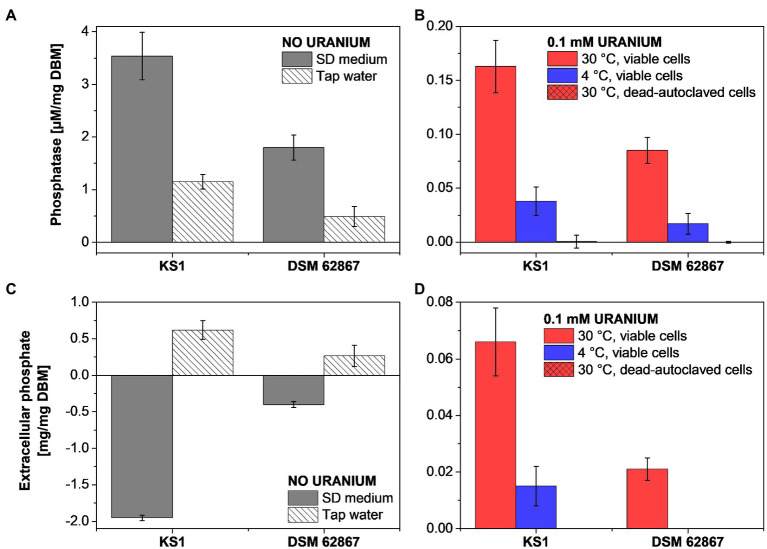
Phosphatase activity **(A,B)** and extracellular phosphate **(C,D)** determination of *P. simplicissimum* KS1 and DSM 62867 after 48 h incubation without U in SD medium or tap water **(A,C)** and with 0.1 mM U in tap water **(B,D)**.

Based on the microscopic data, phosphates appear to be crucially relevant for the U removal *via* active biomineralization by *P. simplicissimum* KS1. Since this might have been mediated by phosphatase activity, the quantification of orthophosphate concentration and phosphatase activity of *P. simplicissimum* KS1 and DSM 62867 were studied in sterile-filtered tap water with an initial U concentration of 0.1 mM and without U in SD medium (Figure 3).

Overall, *P. simplicissimum* KS1 showed a substantially higher phosphatase activity and extracellular orthophosphate concentration than *P. simplicissimum* DSM 62867, independent of the studied media. This pivotal observation is in good agreement with the HAADF-STEM results (Figure 2; Supplementary Figures 6, 7). There, *P. simplicissimum* KS1 demonstrated a higher extracellular amount of phosphorus, overlapping with the U signal. Furthermore, the phosphatase activity decreased with a decreasing amount of nutrients (i.e., in presence of tap water), decreasing temperature, and dead-autoclaved cells. As shown before, such results point to the major role of active metabolic processes for U removal.

In addition, a temperature decline of samples with dead cells led to a decreasing amount of extracellular phosphate. These results are in line with those obtained by HAADF-STEM (Figure 2), where viable *P. simplicissimum* KS1 cells at 30°C showed prominent biomineralization, probably driven by phosphatases, decreasing with temperature declining to 4°C, and not detected with dead-autoclaved cells. The importance of phosphates and phosphatases in the removal of U by bacteria (Beazley et al., 2007; Kulkarni et al., 2016) and the contribution of phosphate transporter genes in U tolerance of *S. cerevisiae* (Sakamoto et al., 2012) have been described in the literature and come to support their observed involvement in U precipitation by *P. simplicissimum* KS1 and DSM 62867.

### Identification of Bio-Associated and Extracellular U Species: Cryo-TRLFS Studies

Cryo-TRLFS was used to investigate the effect of temperature and cell viability on the luminescence properties (i.e., emission bands) of the U species associated with or produced by the cells of the fungal isolate *P. simplicissimum* KS1. To this end, the supernatant and fungal biomass were measured separately after the incubation with 0.1 mM U(VI). Together with the kinetic, microscopic, and spectrophotometric experiments, the obtained data would help to identify processes by which the fungal isolate interacts with the radionuclide.

**Figure 4:**
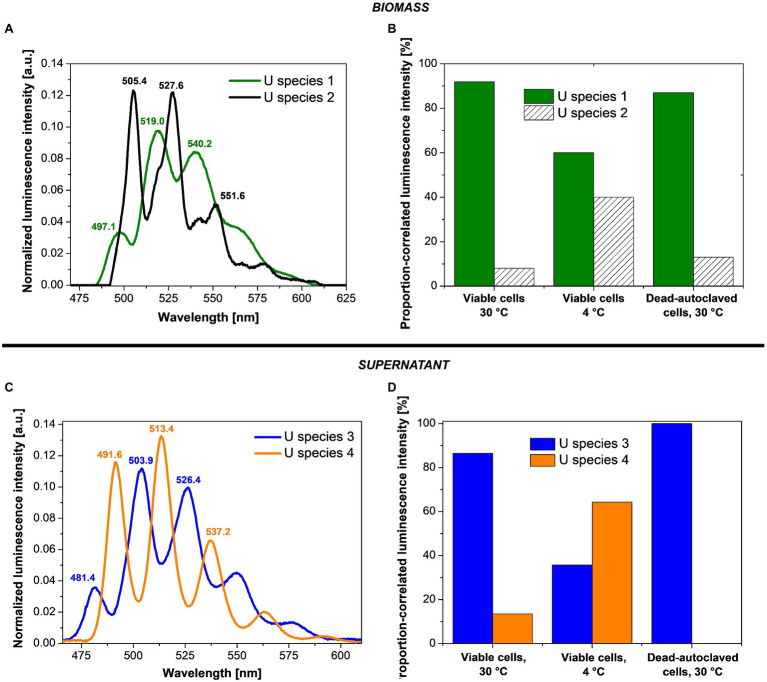
Deconvoluted luminescence spectra **(A,C)** and species distribution **(B,D)** based on the PARAFAC analyses of *P. simplicissimum* KS1 biomass (top) and supernatant (bottom) after 48 h incubation with 0.1 mM U(VI) at pH 5.0.

PARAFAC studies based on cryo-TRLFS spectra of the U-treated fungal biomass (0.1 mM U for 48 h and a fungal DBM of ~0.25 g/L) showed two dominant U(VI) species (Figures 4A,B). At varying temperature (4 and 30°C) and depending on fungal cell viability (viable or dead-autoclaved), the two species were detected in different proportions (Figure 4B). The first species (U species 1, green) dominated all three samples and was characterized by three main emission bands at 497.1, 519.0, and 540.2 nm, as shown in the luminescence spectrum (Figure 4A). With 92% (~64 mg U/g DBM) at 30°C and viable cells, this U species was proportionally and quantitatively more prominent as compared to viable cells at 4°C (60%, ~14 mg U/g DBM) and dead-autoclaved cells at 30°C (87%, ~11 mg U/g DBM). Given this observation and the lower fine structure when compared to the second species, proportionally less-present (U species 2, black), it was assumed that the dominating species represented a bio-associated organic U phosphate species that was produced actively and passively by the fungal cells. The second species, having a greater fine structure and shifted emission bands (505.4, 527.6, and 551.6), was assumed to correspond to a more homogenous, inorganic U(VI) phosphate species (Wang et al., 2008), which could be produced actively by fungal phosphatase activity.

In addition to the two bio-associated U(VI) species detected, two further species (named U species 3 and 4) were calculated *via* PARAFAC in the resulting supernatant after the incubation of *P. simplicissimum* KS1 in 0.1 mM U(VI; Figures 4C,D). U species 3 (Figure 4C, blue) showed emission bands at 481.4, 503.9, and 526.4 nm; U species 4 (Figure 4C, orange) at 491.6, 513.4, and 537.2 nm. Both species were more homogenous than the bio-associated species 1. As for the bio-associated species, U species 3 and 4 were detected in different proportions depending on cell viability and temperature (Figure 4D). U species 3 was dominant in the supernatant of dead-autoclaved cells (100%) and viable cells at 30°C (87%), but at 4°C, U species 4 (64%) surpassed species 3 (36%). U species 4 was only present to 10% in viable cells at 30°C and not detectable in the supernatant of dead-autoclaved cells at 30°C. Thus, U species 4 was most likely only secreted actively by viable cells, displaying increased proportions at low temperature (4°C). Remarkably, uranyl nitrate, the initially added uranium species, was not observed. This indicates that all the uranium in solution interacted with biological matter.

Identification of the exact uranyl species based on the emission bands of reference compounds is difficult due to varying experimental conditions and thus different complexing agents and resulting spectral shifts. All four species can be assigned to U(VI) phosphate species of organic or inorganic origin in view of their characteristic spectral shapes. Accordingly, these species in particular could also be actively produced by fungal phosphatase activity. A comparison of the emission bands obtained in the present work with those described in the literature (Table 1) shows U species 1 to share characteristic emission bands with organic phosphate ligands, e.g., adenosine-monophosphate, fructose(6)-phosphate, or lipopolysaccharides at the cell wall through biosorption (Merroun et al., 2003; Barkleit et al., 2004, 2008). The bio-associated inorganic uranyl phosphate species (U species 2) showed emission bands similar to those of autunite (Geipel et al., 2000), as previously reported for various fungal species, and could resemble the needle-shaped uranyl phosphate structures in Figure 2 (Günther et al., 2014; Gerber et al., 2018; Lopez-Fernandez et al., 2018). Furthermore, the supernatant U species 3 and 4 revealed emission bands approximately matching those of other organic ligands – i.e., phospholipids (phosphocholine and phosphoserine) and phosphorylated amino acids (threonine, tyrosine, and tryptophan; Günther et al., 2006; Koban and Bernhard, 2007; Wollenberg et al., 2021). An involvement of organic uranyl species in fungal U removal was described for the acid-tolerant fungus *Coniochaeta fodinicola* (Vázquez-Campos et al., 2015). Contrary to *P. simplicissimum* KS1, the dead-autoclaved *Coniochaeta* biomass removed more U (~45 mg U/g DBM) when compared to viable cells (16 mg U/g DBM; Vázquez-Campos et al., 2015). The authors concluded that phosphates, polysaccharides, and organic acids were released after cell death, hence were not available for U sorption in the case of viable cells (Vázquez-Campos et al., 2015). Recently, Wollenberg et al. (2021) observed *via* TRLFS that phosphorylated amino acids, released by fungal species, interacted with uranium. In the fungus *Schizophyllum commune*, tryptophan and related indole derivatives, related to the emission bands of the only actively secreted U(VI) species 4, could act as messenger molecules in the stress response of *S. commune* (Wollenberg et al., 2021). The suggested scenarios, in combination with active release of organic ligands before cellular death, could explain the higher U removal by viable *P. simplicissimum* KS1 cells than by dead-autoclaved cells (Figure 1) and is supported by the superimposed EDXS signals of phosphorus and U (Figure 2) and increased extracellular orthophosphate concentration after U exposure (Figure 3).

**Table 1:** Luminescence emission bands of the two determined U(VI) species of *Penicillium simplicissimum* KS1 cells exposed to 0.1 mM U(VI) compared to band positions of reference spectra of organic uranyl phosphate species.

	Luminescence emission bands (nm)			References
Bio-associated U(VI) species 1, pH 5.0	497.1	519.0	540.2	This work
UO_2_-adenosine monophosphate	497	519	542	Merroun et al., 2003
UO_2_-fructose(6)-phosphate	497.1	519.0	543.3	Barkleit et al., 2004
UO_2_-PO_3_-O-R (Lipopolysaccharides)	498.1	519.6	542.9	Barkleit et al., 2008
Bio-associated U(VI) species 2, pH 5.0	505.4	527.6	540.2	This work
Autunite	504.0	524.2	548.0	Geipel et al., 2000
Supernatant U(VI) species 3, pH 5.0	481.4	503.9	526.4	This work
UO_2_-phosphocholine	481.1	497.2	517.6	Koban and Bernhard, 2007
UO_2_-phosphoserine	482.0	496.2	516.5	Koban and Bernhard, 2007
UO_2_-HP-threonine	483.7	501.8	523.4	Günther et al., 2006
Supernatant U(VI) species 4, pH 5.0	491.6	513.4	537.2	This work
U(VI)-tryptophan	490	510	530	Wollenberg et al., 2021
U(VI)-phosphotyrosine	492	515	539	Wollenberg et al., 2021
U(VI)-phosphothreonine	494	515	537	Wollenberg et al., 2021

Error of emission bands: ±0.5 nm.

### Hypothetical Interaction Mechanism of *P. simplicissimum* KS1 With U(VI)

To sum up, cell viability and temperature critically influence the interaction of the fungus *P. simplicissimum* KS1 with U(VI). Through kinetic experiments (Figure 1), EDXS-based spectrum imaging analysis (Figure 2), fluorometric and chromatographic analyses of phosphatase and orthophosphate (Figure 3), and TRLFS studies (Figure 4), these parameters were investigated, their results being summarized in Supplementary Table 4 and pictured in the Graphical Abstract.

By studying the parameters cell viability and temperature, it was concluded that *P. simplicissimum* KS1 removes U(VI) actively from solution at 30°C, mainly *via* extracellular biomineralization, aside from minor biosorption and bioaccumulation. At 4°C with viable cells and after incubation with dead-autoclaved cells at 30°C, less U(VI) was removed (and slower at 4°C), thus indicating a key role of metabolic processes in heavy-metal interaction. The uranium precipitations were mainly identified extracellularly, again decreasing in amount when experimental conditions changed to lower temperatures or dead-autoclaved cells. Therefore, the extracellular U(VI) precipitations were mainly produced actively *via* biomineralization. Phosphatase activity and extracellular orthophosphate concentration were moreover decreased, further supporting the hypothesis of active biomineralization driving U(VI) removal from solution. Ultimately, the U speciation studies revealed organic and inorganic U(VI) phosphates and additional organic ligands (i.e., phosphorylated amino acids), which could act as actively secreted messenger molecules.

## Conclusion

The results presented here highlight the potential of the heavy metal-adapted fungal isolate *P. simplicissimum* KS1 for bioremediation of U- and other heavy-metal-contaminated sites. The elevated U removal capacity of the fungal isolate is compared to that of the non-U-adapted reference strain *P. simplicissimum* DSM 62867. Although, intra- and extracellular U accumulations are observable for both strains, the extracellular U precipitations are greater for *P. simplicissimum* KS1. Electron microscopy, TRLFS and ICP-MS studies revealed a temperature- and cell viability-dependent U biomineralization, thus indicating its dependency on active cell metabolism. Compared to viable *P. simplicissimum* KS1 cells at 30°C, a decrease in temperature to 4°C or the incubation with dead-autoclaved cells at 30°C decreased extracellular biomineralization, which was replaced by passive biosorption and bioaccumulation. Bio-associated U species were mainly assigned to uranyl phosphates by EDXS and TRLFS. Additionally, the fungus secreted small phosphorylated amino acids, driven by temperature and cell viability, that interacted with U and could act as messenger molecules. Our outcomes demonstrate not only the efficient removal of U from solutions by *P. simplicissimum* KS1, hence its potential for the bioremediation of U-contaminated sites, but also the key role of temperature and cell viability in terms of metabolic influence on the interaction of the fungus with U.

For the bioremediation of U-contaminated waters, as in the former U mine in Königstein (Germany), *P. simplicissimum* KS1 might be a candidate to support cost- and time-intensive chemical treatment. Since this fungal strain is present in the flooding water already, sub-surface systems enriching the fungus by the addition of an environmental-friendly carbon source (e.g., glucose or fructose) are imaginable, as well as aboveground systems exploiting the available pumps and engines.

Prospectively, further experiments are needed to evaluate the U removal capacity of the fungal isolate for *in-situ* bioremediation on the industrial scale. In addition, U recovery experiments should be performed.

## Data Availability Statement

The original contributions presented in the study are included in the article/Supplementary Material; further inquiries can be directed to the corresponding authors.

## Author Contributions

SS carried out the experiments, wrote the original draft of the manuscript with support from RS, RH, EK-B, MM, and edited it after the internal revisions. RS introduced SS into cryo-TRLFS measurements and performed the computational analysis *via* PARAFAC. RH conducted the electron microscopy experiments and provided critical feedback to the project. EK-B and MM supervised the project, helped in interpreting the results, and reviewed the manuscript. All authors contributed to the article and approved the submitted version.

## Funding

This work was supported by the Bundesministerium für Bildung und Forschung (BMBF) grant no. 02NUK030 F (TransAqua). Funding of TEM TALOS at the HZDR Ion Beam Center TEM facilities by the German Federal Ministry of Education and Research (BMBF grant no. 03SF0451) in the framework of HEMCP is acknowledged. The open-access publication fees were kindly covered by the library of the Helmholtz-Zentrum Dresden-Rossendorf (Germany). SS was partially supported during his research stay in Granada (Spain) by the Talent Acquisition Program (“Programa de Captación de Talento en Grados Universitarios”), funded by the University of Granada (Spain).

## Conflict of Interest

The authors declare that the research was conducted in the absence of any commercial or financial relationships that could be construed as a potential conflict of interest.

## Publisher’s Note

All claims expressed in this article are solely those of the authors and do not necessarily represent those of their affiliated organizations, or those of the publisher, the editors and the reviewers. Any product that may be evaluated in this article, or claim that may be made by its manufacturer, is not guaranteed or endorsed by the publisher.

## Footnotes

^1^https://blast.ncbi.nlm.nih.gov/Blast.cgi (Accessed May 18, 2017).
