# Supplementary material for: Effect of Temperature and Cell Viability on Uranium Biomineralization by the Uranium Mine Isolate Penicillium simplicissimum
Source: Front Microbiol. 2021 Dec 22;12:802926. doi: 10.3389/fmicb.2021.802926 (PMC8728092; doi:10.3389/fmicb.2021.802926)
Supplement: Supplementary file 1 [file Data_Sheet_1.pdf]

# - *Supplementary Material* -

Effect of temperature and cell viability on uranium biomineralization by the uranium mine isolate *Penicillium simplicissimum*

Sebastian Schaefer[1,a*], Robin Steudtner[1], René Hübner[2], Evelyn Krawczyk-Bärsch[1*], Mohamed L. Merroun[3]

[1]Institute of Resource Ecology, Helmholtz-Zentrum Dresden-Rossendorf, Dresden, Germany

[2]Institute of Ion Beam Physics and Materials Research, Helmholtz-Zentrum Dresden-Rossendorf, Dresden, Germany

[3]Department of Microbiology, University of Granada, Granada, Spain

*** Correspondence:**
Evelyn Krawczyk-Bärsch - e.krawczyk-baersch@hzdr.de
Sebastian Schaefer – s.schaefer@unsw.edu.au

[a] Current address: School of Chemical Engineering, University of New South Wales, Sydney, New South Wales, Australia

Table of contents

1 Supplementary results 3

1.1 Fungal growth dependence on different carbon sources 3

1.2 Heavy metal tolerance of *P. simplicissimum* KS1..4

1.3 *P. simplicissimum* KS1 cell viability in tap water (+ 0.1 mM uranium) 5

1.4 Uranium removal capacity depending on fungal dry biomass 6

1.5 Comparison of maximum U removal capacities of selected fungal species 7

1.6 SEM results of *P. simplicissimum* KS1 and DSM 62867 8

1.7 Additional spectrum imaging results of *P. simplicissimum* KS1 and DSM 62867 10

1.8 Summary of experimental results of *P. simplicissimum* KS1 13

2 Supplementary experimental section..14

2.1 Fungal growth dependence on carbon sources 14

2.2 Determination of tolerance towards heavy metals of *P. simplicissimum* KS1..............14

2.3 Cell viability of *P. simplicissimum* KS1 determined by plate counting.......................14

3 Additional references...15

1 Supplementary results

1.1 Fungal growth dependence on different carbon sources

Supplementary Table 1 - Growth of *P. simplicissimum* KS1 in different carbon sources (1 % w/v each). Growth was examined visually (+++: good growth, ++: medium growth, +: poor growth, -: no growth).

**Carbon source**	***P. simplicissimum* KS1**
**Ethanol**	-
**Fructose**	+++
**Galactose**	++
**Glucose**	+++
**Glycerol**	+
**Lactate**	-
**Maltose**	+
**Mannose**	++
**Oxalic acid**	-
**Saccharose**	++
**Sodium acetate**	-
**Xylose**	++

1.2 Heavy metal tolerance of *P. simplicissimum* KS1

Supplementary Table 2 – Tolerance of *P. simplicissimum* KS1 against selected heavy metals in 1:5 diluted SD medium.

**Heavy metal**	***P. simplicissimum* KS1**<br>**MIC [mM]**
**Cadmium**	2.0
**Chromium**	> 22.0
**Cobalt**	1.0
**Copper**	1.0
**Lead**	5.0
**Manganese**	1.0
**Nickel**	0.2
**Uranium**	0.7
**Zinc**	> 15.0

1.3 *P. simplicissimum* KS1 cell viability in tap water (+ 0.1 mM uranium)

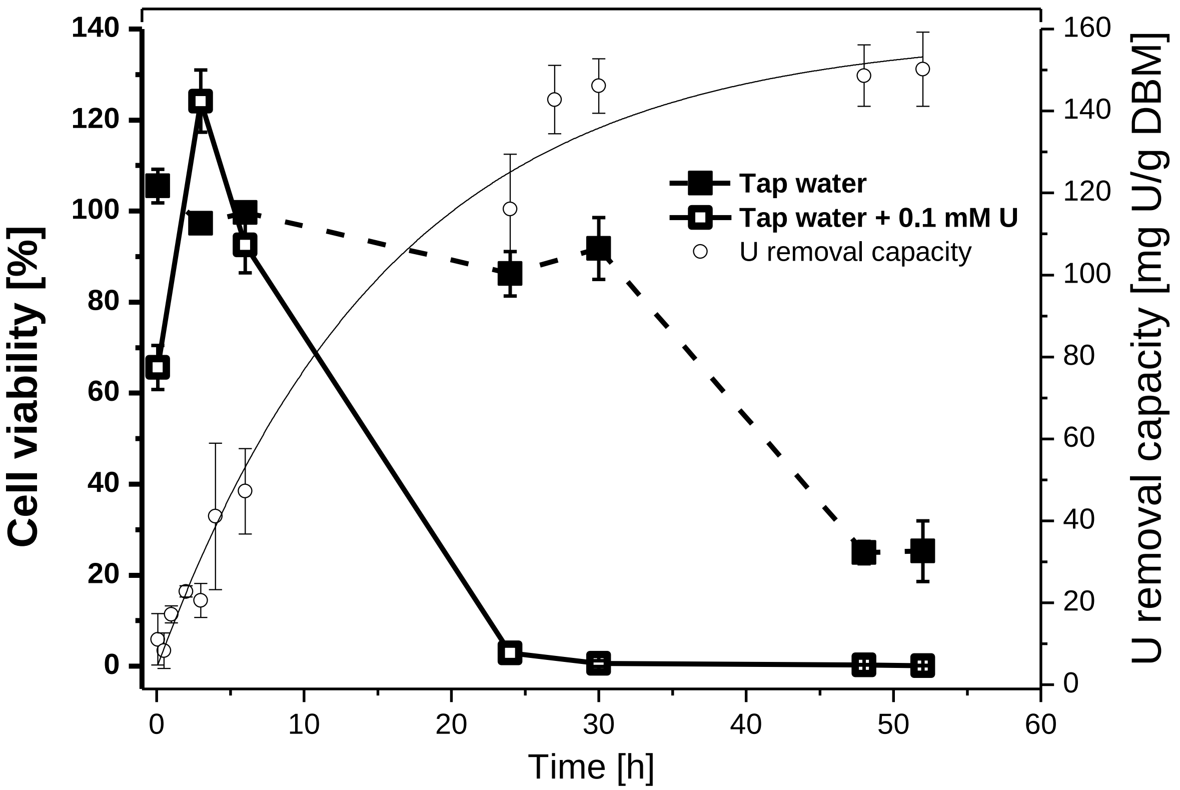

Supplementary Figure 1 – *P. simplicissimum* KS1 cell viability in sterile-filtered tap water (black marks, dotted line) and 0.1 mM uranium (empty marks, solid line) over 52 h at 30 °C. The uranium removal capacity is shown (empty, round marks) with its corresponding exponential fit (solid, thin line). Standard deviations are depicted as error bars.

1.4 Uranium removal capacity depending on fungal dry biomass

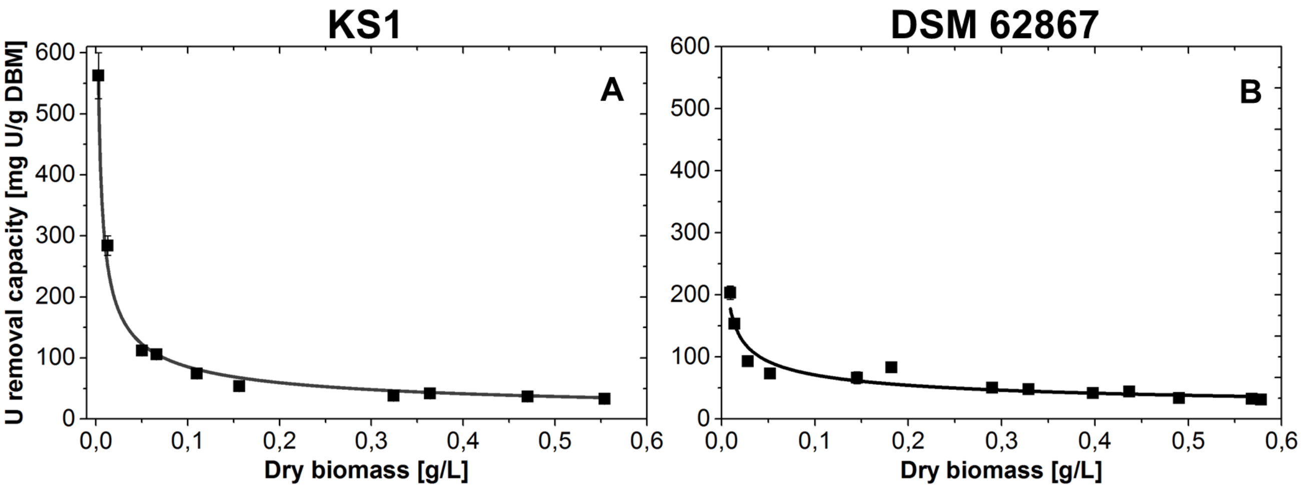

Supplementary Figure 2 – Uranium removal capacity of *P. simplicissimum* KS1 (A) and DSM 62867 (B) depending on dry biomass after 48 h at 30 °C and 0.1 mM uranium. The lines correspond to their respective exponential fits. Standard deviations are depicted as error bars.

1.5 Comparison of maximum U removal capacities of selected fungal species

Supplementary Table 3 – Maximum U removal capacities of selected fungal species. [1] Liu *et al.*, 2010, [2] Treen-Sears *et al.*, 1984, [3] Gerber *et al.*, 2018

**Species**	**Maximum U removal capacity (mg U/g DBM)**	**Reference**
Penicillium simplicissimum KS1	550	This work
Saccharomyces cerevisiae	102	[1]
Rhizopus sp.	260	[2]
Rhodosporidium toruloides	350	[3]

1.6 SEM results of *P. simplicissimum* KS1 and DSM 62867

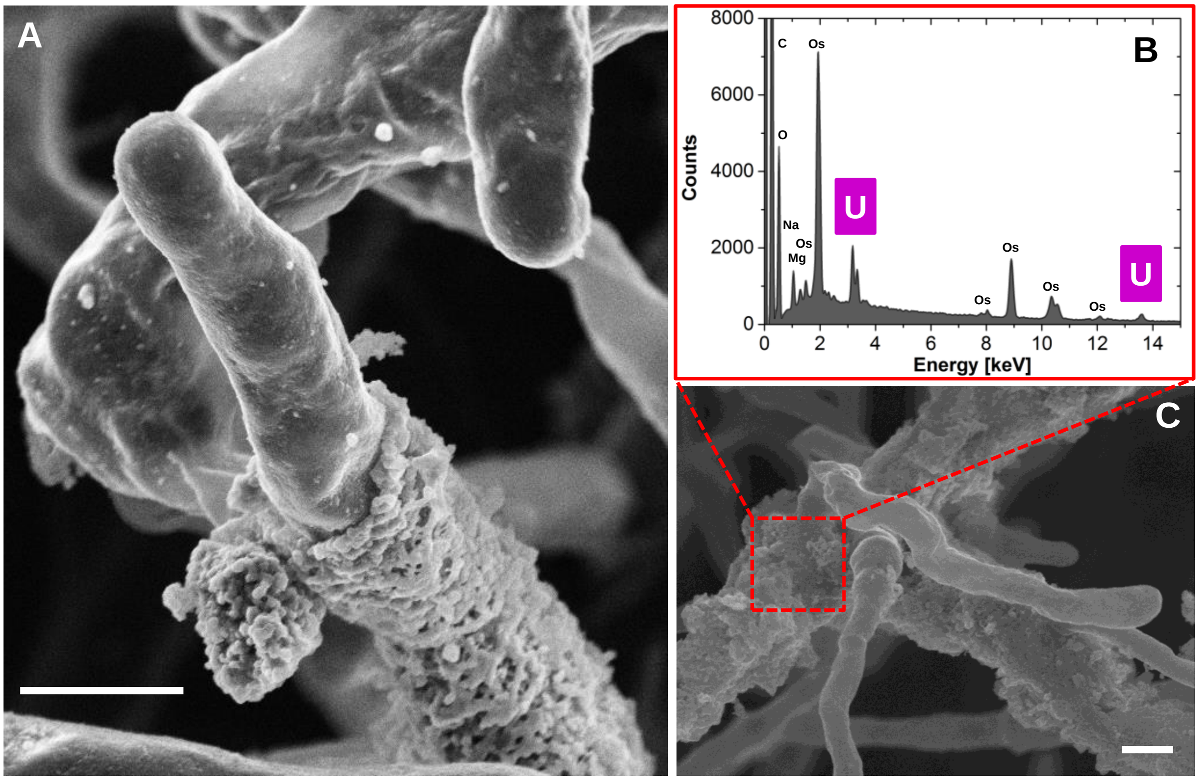

Supplementary Figure 3 – SEM images (A and C) of *P. simplicissimum* KS1 after incubation for 48 h in 0.1 mM uranium (background: sterile-filtered tap water pH 5.0) at 30 °C. The EDXS measurement (B) of extracellular accumulations (C) indicates uranium. The scale bars represent 2 µm.

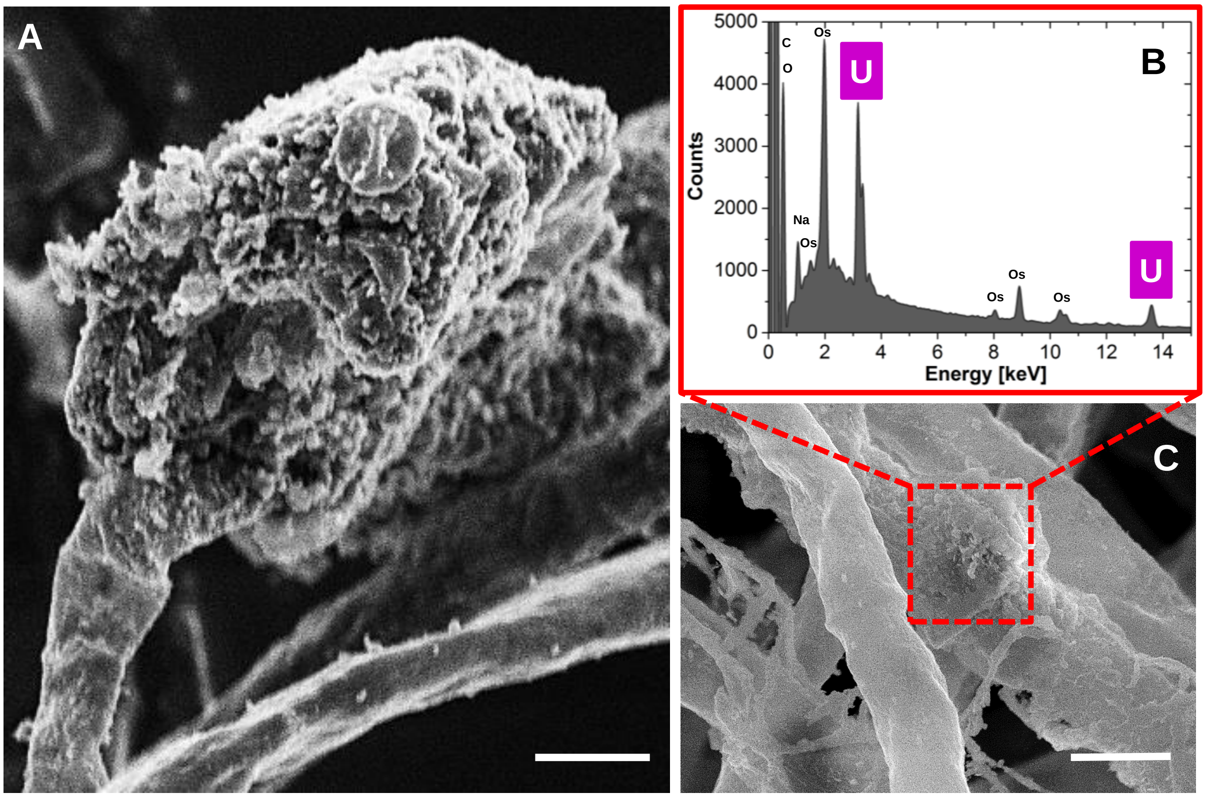

Supplementary Figure 4 – SEM images (A and C) of *P. simplicissimum* DSM 62867 after incubation for 48 h in 0.1 mM uranium (background: sterile-filtered tap water pH 5.0). The EDXS measurement (B) of extracellular accumulations (C) indicates uranium. The scale bars represent 2 µm.

Note: Phosphorus was not significantly visible in the EDX spectra of the SEM measurements, since its characteristic Kα signal (2.013 keV) coincides with the M lines of osmium (1.914 keV), which was used during the preparation in order to receive an increased sample contrast. However, with the help of computational analysis (peak deconvolution) and the known ratio of osmium signal intensities, the presence of phosphorus can be determined.

1.7 Additional spectrum imaging results of *P. simplicissimum* KS1 and DSM 62867

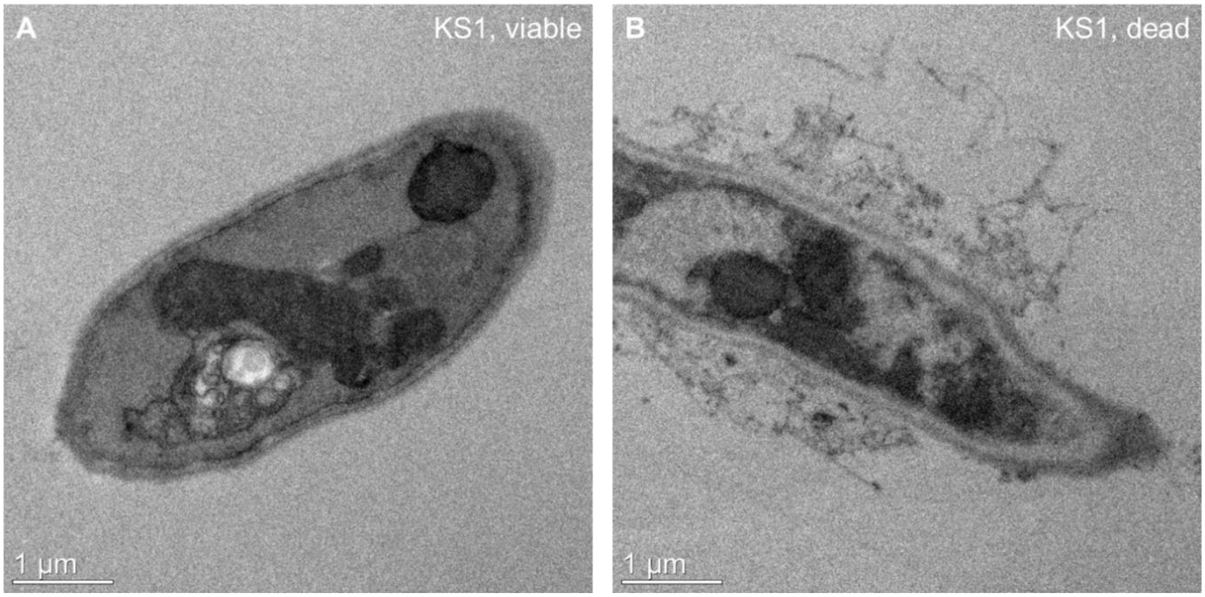

Supplementary Figure 5 – Bright-field TEM micrographs of *P. simplicissimum* KS1 without uranium comparing viable (A) and dead-autoclaved cells (B) after 48 h growth in SD medium.

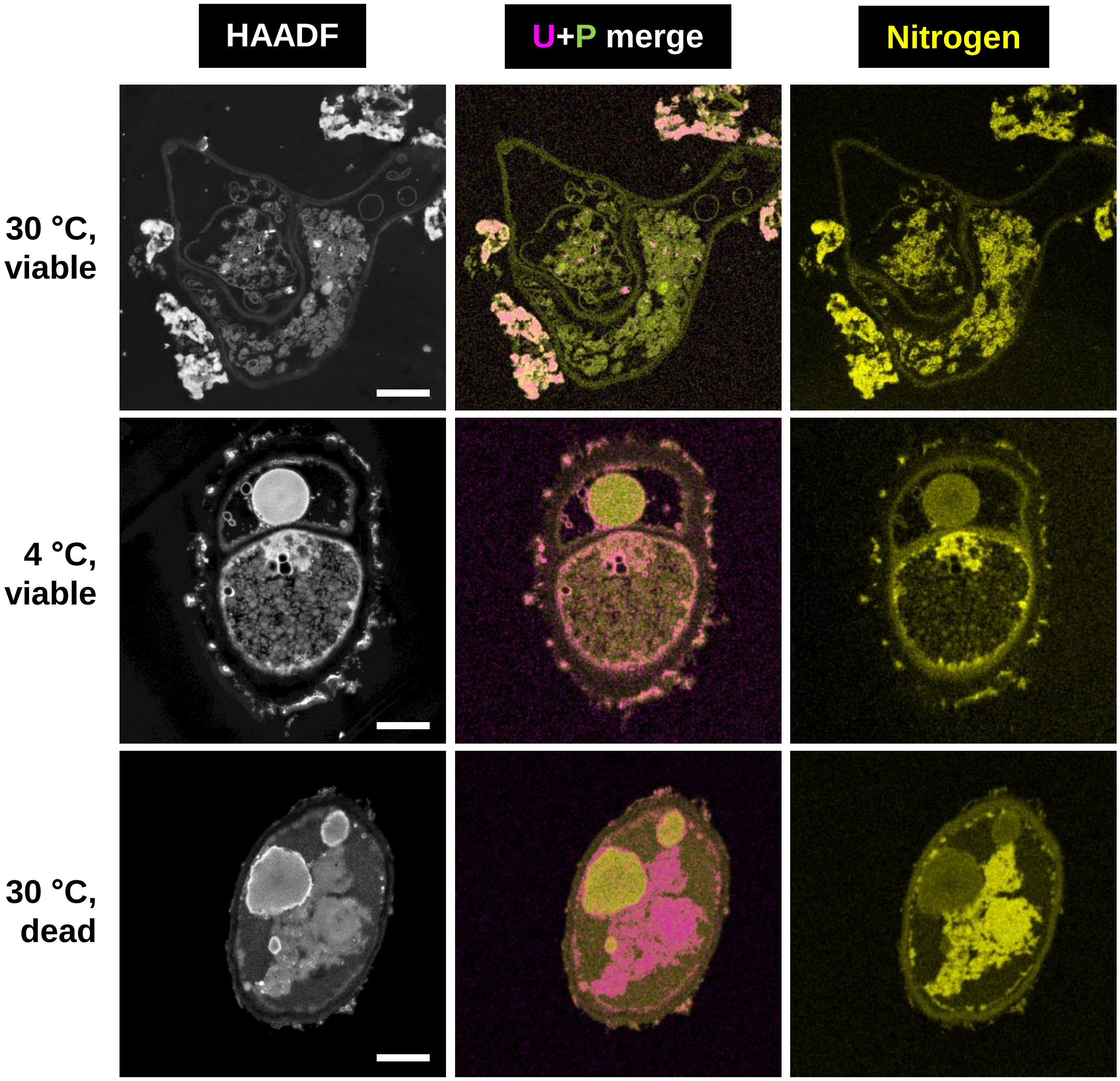

Supplementary Figure 6 – HAADF-STEM micrographs of viable *P. simplicissimum* KS1 at 30 °C and 4 °C (top and center rows) and dead-autoclaved cells at 30 °C (bottom row) together with EDXS-based element distributions (magenta: uranium; green: phosphorus; yellow: nitrogen). The fungal isolate was incubated in 0.1 mM uranium (background electrolyte: sterile-filtered tap water pH 5.0) for 48 h. The scale bars indicate 1 µm.

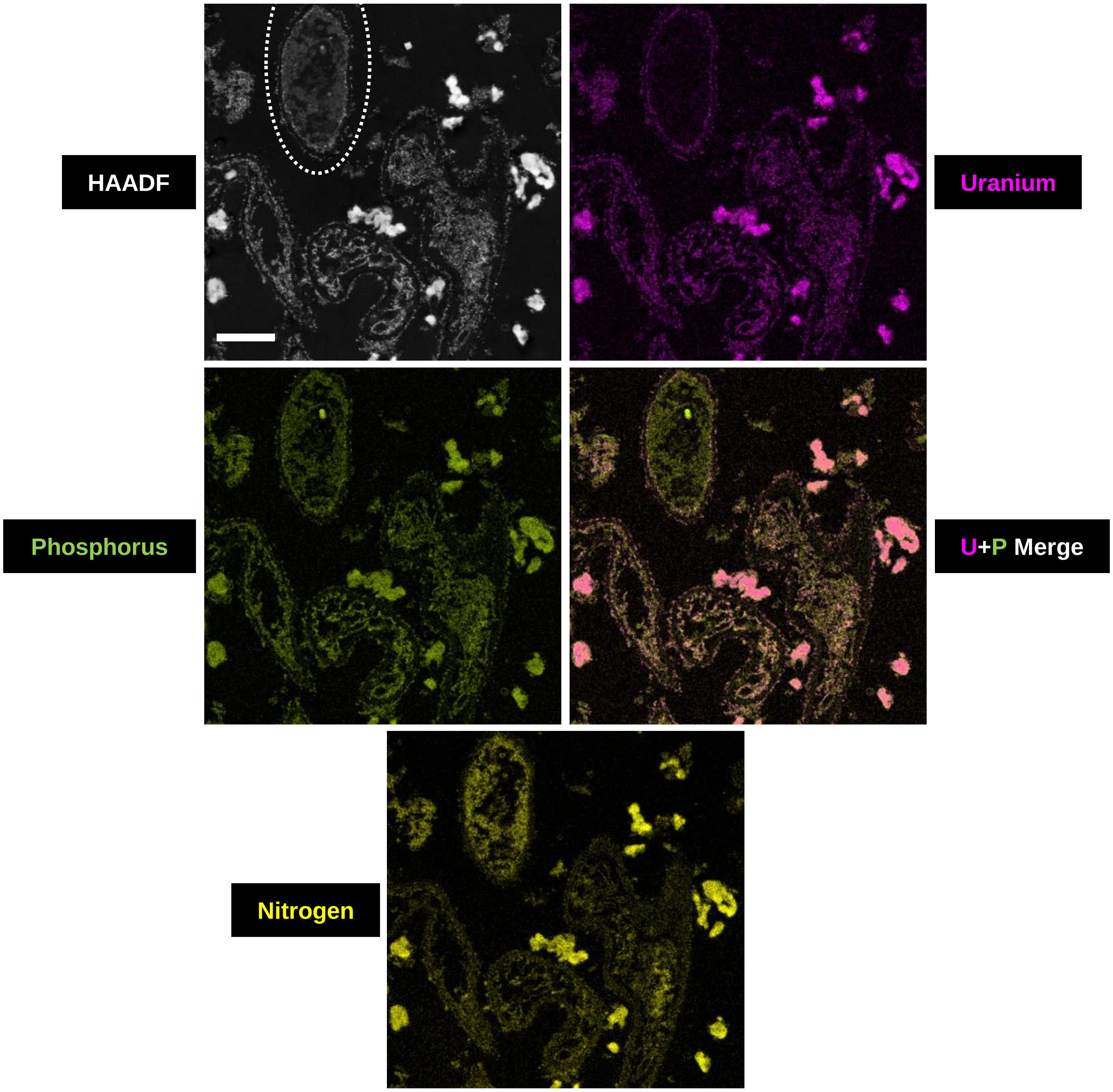

Supplementary Figure 7 - HAADF-STEM micrograph of *P. simplicissimum* (DSM 62867) together with EDXS-based element distributions for uranium (magenta), phosphorus (green), and nitrogen (yellow). The dotted oval ellipse highlights a putatively viable cell since no intracellular uranium was detected. DSM 62867 was incubated in 0.1 mM uranium (background: sterile-filtered tap water pH 5.0) for 48 h at 30 °C. The scale bar indicates 1 µm.

1.8 Summary of experimental results of *P. simplicissimum* KS1

Supplementary Table 4 – Outcomes of viable and dead-autoclaved *P. simplicissimum* KS1 cells after incubation in 0.1 mM U(VI) for 2 days and at respectively comparable dry biomasses.

		Viable cells		Dead-autoclaved cells
		**30 °C**	**4 °C**	**30 °C**
**U removal capacity (mg U/g DBM) ***		107	26	34
**Time to reach maximum U removal**		~24 h	~30 h	0 h
**Localization of U precipitations**		Majority extracellularly, minority intracellularly	Extra- and intracellularly	Majority intracellularly, minority extracellularly
**EDXS-based U speciation**		Superposition of U with phosphorus and nitrogen		
**Phosphatase (µM/mg DBM)**		0.16	0.04	0.00
**Extracellular orthophosphate (mg/mg DBM)**		0.066	0.015	0.000
**Bio-associated U species**	**Organic ligands**	92%	60%	87%
	**Anorganic ligands**	8%	40%	13%
**Supernatant U species**		Phospholipids and phosphorylated amino acids	Phospholipids and phosphorylated amino acids	Phospholipids and amino acids (only passive interaction)
**Suggested processes**		Biosorption, bioaccumulation and biomineralization (majority)	Biosorption, bioaccumulation and biomineralization (more equally)	Biosorption

Note: * at 0.1 g DBM/L

2 Supplementary experimental section

2.1 Fungal growth dependence on carbon sources

The fungal growth of *P. simplicissimum* KS1 was studied depending on different carbon sources. Ethanol, fructose, galactose, glucose, glycerol, lactate, maltose, mannose, oxalic acid, saccharose, sodium acetate and xylose (Carl Roth) were tested. The carbon source (10 % w/v stock solutions) was added to 20 mL of minimal salt medium (MSM; 20 mg/L $K(HPO_4)_2$, 60 mg/L K_2HPO_4, 50 mg/L $(NH_4)_2SO_4$, 10 mg/L $MgSO_4$, 1 mg/L $CaCl_2$, 0.5 mg/L $FeCl_3$, 10 mg/L $MnCl_2$, final pH: 5.2, Carl Roth) to a final concentration of 1 % w/v. 50 µL of washed cells were added. Therefore, the cells were sterile-filtered (qpore® sterile membrane filter, 0.22 µm pore-size; neoLab Migge) and washed with MSM twice. After incubation over 48 h, 30 °C and 130 rpm the fungal growth was evaluated macroscopically by comparison with the control sample. The experiments were performed in duplicates.

2.2 Determination of tolerance towards heavy metals of *P. simplicissimum* KS1

The fungal growth of *P. simplicissimum* KS1 was studied depending on the heavy metal concentration. Cadmium (0.01 – 2.0 mM), chromium (0.2 – 22.0 mM), cobalt (0.1 – 5.0 mM), copper (0.05 – 0.8 mM), lead (0.2 – 3.0 mM), manganese (0.1 - 5.0 mM), nickel (0.1 – 5.0 mM), uranium (0.05 mM – 10.0 mM) and zinc (0.2 – 15.0 mM) were tested using their dissolved nitrate compounds (Carl Roth). To this end, the heavy metal (1 M stock solutions except for uranium 0.2 M stock solution) was added to 1:5 diluted SD medium. Afterwards 50 µL of washed fungal cells were spread out on solid agar plates and incubated for 72 h at 30 °C. The fungal growth, especially the MIC, was evaluated macroscopically. All experiments were performed in duplicates.

2.3 Cell viability of *P. simplicissimum* KS1 determined by plate counting

To investigate the cell viability of *P. simplicissimum* KS1 depending on the medium (sterile-filtered tap water, pH= 5.0) with or without addition of 0.1 mM $UO_2(NO_3)_2$, fungal cells were prepared as described in the main materials and methods section. 50-µL samples were taken in triplicates after 0, 4, 7, 24, 30, 48, and 52 h and spread out on individual SD plates. The plates were incubated for 48 h at 30 °C. The vegetated surface was compared to the average of three control plates (each 50 µL fungal cells on SD plates, incubated for 48 h at 30 °C) by using the open-source software Fiji (Schindelin *et al.*, 2012).

3 Additional references

Gerber, U., Hübner, R., Rossberg, A., Krawczyk-Bärsch, E., and Merroun, M.L. (2018). Metabolism-dependent bioaccumulation of uranium by *Rhodosporidium toruloides* isolated from the flooding water of a former uranium mine. *PLoS One* 13**,** e0201903.

Liu, M., Dong, F., Yan, X., Zeng, W., Hou, L., and Pang, X. (2010). Biosorption of uranium by *Saccharomyces cerevisiae* and surface interactions under culture conditions. *Bioresour. Technol.* 101**,** 8573-8580.

Schindelin, J., Arganda-Carreras, I., Frise, E., Kaynig, V., Longair, M., Pietzsch, T., Preibisch, S., Rueden, C., Saalfeld, S., Schmid, B., Tinevez, J.-Y., White, D.J., Hartenstein, V., Eliceiri, K., Tomancak, P., and Cardona, A. (2012). Fiji: an open-source platform for biological-image analysis. *Nat. Methods* 9**,** 676-682.

Treen-Sears, M.E., Volesky, B., and Neufeld, R.J. (1984). Ion exchange/complexation of the uranyl ion by *Rhizopus* biosorbent. *Biotechnol. Bioeng.* 26**,** 1323-1329.
